# The influence of feminist abortion accompaniment on emotions related to abortion: A longitudinal observational study in Mexico

**DOI:** 10.1016/j.ssmph.2022.101259

**Published:** 2022-10-04

**Authors:** Alexandra Wollum, Sofía Garduño Huerta, Oriana López Uribe, Camille Garnsey, S. Michael Gaddis, Sarah E. Baum, Brianna Keefe-Oates

**Affiliations:** a Ibis Reproductive Health, Oakland, CA, USA; b Department of Community Health Sciences, Fielding School of Public Health, University of California, Los Angeles, USA; c Balance, Mexico City, Mexico; d Department of Sociology, University of California, Los Angeles, USA

**Keywords:** Abortion, Accompaniment, Mexico, Emotions, Abortion stigma, Person-centered care

## Abstract

Emotions can reflect how individuals internalize identities, social roles, and broader power structures, including abortion stigma. Abortion accompaniment, in the form of logistical, informational, and emotional support offered by individuals and organizations, takes a person-centered, feminist, and rights-based approach. We tested the extent to which abortion accompaniment may decrease negative and increase positive feelings an individual holds related to their abortion. Using observational longitudinal data collected between January 2017 and mid-2018, we compared negative and positive emotional responses to a personal abortion experience one month and six months following the abortion to emotions immediately prior to the abortion (“baseline”), among women travelling from outside of Mexico City to abortion clinics in Mexico City with and without support of the accompaniment organization, Fondo MARIA. We used doubly robust longitudinal mixed effects models with inverse probability weighting methods. At baseline, accompanied and unaccompanied participants experienced an average of 4.9 and 4.4 negative emotions out of eight respectively (i.e., anguish, nervousness, scared, anxious, sadness, guilt, anger, shame) and 1.7 and 1.9 positive emotions of out 4 respectively (happiness, calmness, decidedness, and relief). From our model results, women accompanied (n = 77) had larger decreases in negative feelings (p < .05) and larger increases in positive feelings (p < .01) toward their abortion compared to those who were not accompanied (n = 119) at six months. These changes led the majority of accompanied respondents to have primarily positive feelings about their abortion by endline. Abortion accompaniment through Fondo MARIA in Mexico City was associated with a larger decrease in negative feelings, particularly those related to stigma, and a larger increase in positive feelings six months after abortion. Accompaniment's focus on person-centered support, self-determination, and autonomy may enable people seeking abortion to view their decision as one that is valid and legitimate, and resist the predominant stigmatizing narratives framing abortion as something that is transgressive.

## Highlights

• Abortion accompaniment (i.e., logistical, informational, and emotional support through the abortion process), takes a person-centered, feminist, and rights-based approach.
• We tested whether abortion accompaniment was associated with decreases in negative and increases in positive feelings someone holds related to their abortion.
• Abortion accompaniment was associated with a decrease in negative feelings (particularly those related to stigma) and an increase in positive feelings six months after abortion.
• Accompaniment’s focus on person-centered support and self-determination may help people view their decision as valid and legitimate and resist stigmatizing narratives.
• Additional work should test interventions or approaches to further support dislodging feelings related to abortion-related stigma

## 1 Introduction

While research has documented that the vast majority of people experience relief in the years following their abortion (Broen et al., 2005; Rocca et al., 2015, 2020), some people do experience negative emotions following their abortions. Internalized stigma, defined as negative views people hold toward themselves resulting from “acceptance of negative culture valuations of abortion”, can manifest as shame, guilt, and other negative emotions (Cockrill & Nack, 2013). People seeking abortion may also perceive that others may look down on them following an abortion (perceived stigma) or have experienced discrimination in the process of seeking abortion (enacted stigma), resulting in negative emotions (Rocca et al., 2020). Thus, negative emotions need not stem from the abortion itself, but from internalizing, perceiving, and/or experiencing societal and community disapproval of abortion (APA Task Force on Mental Health and Abortion, 2008; National Academies of Sciences, Engineering, and Medicine et al., 2018; Rocca et al., 2020, 2015). Negative emotions are distinct from clinically significant mental disorders, and a range of emotions are expected in response to any significant (and in this case stigmatized) health event, including a stigmatized event such as abortion (APA Task Force on Mental Health and Abortion, 2008; National Academies of Sciences, Engineering, and Medicine et al., 2018). Yet, emotions contribute to an individual's wellbeing, and therefore are important outcomes in their own right. Moreover, emotions can also be a mechanism through which abortion stigma is operationalized and perpetuated. As such, interventions to reduce negative emotions related to abortion may in turn reduce abortion stigma and its consequences.

While past research has linked post-abortion emotions to conceptual domains of stigma at the individual level (Rocca et al., 2020), some emotions people hold toward abortion are not rooted in stigma (Kimport, 2012; Kumar, 2013; Millar, 2020). For instance, the relationship with the person involved in the pregnancy or feelings related to the pregnancy itself may cause specific emotions that are unrelated to the stigma of abortion (Smith et al., 2016). Furthermore, abortion for many people is a positive and uncomplicated event (Rocca et al., 2020), counter to the social expectation that abortion is emotionally complicated (Kimport, 2012). Whether emotional responses to abortion are linked to stigma and social norms surrounding abortion, social support, or difficulty related to decision making regarding the pregnancy, it is important to understand how to best support people seeking abortion care emotionally; yet little is known about how programs or interventions can improve emotional wellbeing of people seeking services (Rocca et al., 2020).

Community-oriented feminist organizations and individuals committed to defending and promoting sexual and reproductive rights have established a model of abortion support referred to as abortion accompaniment (Bercu et al., 2021; Drovetta, 2015; Gerdts & Hudaya, 2016; Veldhuis et al., 2022; Zurbriggen et al., 2018). While the accompaniment model varies from organization to organization and between accompaniers, the accompaniment model broadly includes providing support, evidence-based information, and often helping people safely access and use medication abortion outside of the health sector (Drovetta, 2015; Erdman, Jelinska, & Yanow, 2018; Moseson et al., 2022). Accompaniment organizations differ in the methods they use to communicate with abortion-seekers (e.g., phone, text, in-person) and the types of support they provide (e.g., financial, logistical, emotional etc.). They aim to decrease barriers to safe abortion services and empower individuals with the information and support they need to make informed decisions about their abortion and choices related to the abortion process (Berro Pizzarossa & Nandagiri, 2021). The MARIA Abortion Fund for Social Justice (Fondo MARIA) is an abortion accompaniment fund operated by Balance, an organization based in Mexico City (CDMX) dedicated to promoting and defending the reproductive and sexual rights of women^1^ and young people. Fondo MARIA supports people across Mexico seeking abortions using an “accompany to empower” model, providing information and support to help individuals who call their hotline make an informed decision about whether they would prefer to access clinic services in CDMX,^2^ or self-manage a medication abortion at home. The organization provides additional logistical, information, emotional, and financial support to help them do either. Between May 2009 and May 2020, Fondo MARIA helped over 10,500 people in Mexico access abortion services (Fondo MARIA, 2020).

Through taking a person-centered, feminist, and rights-based approach, accompaniment has the potential to transform the emotional experience for people as they navigate the abortion process. In this paper, we assess whether abortion accompaniment from Fondo MARIA influences the emotions that individuals feel related to their abortion. We compare experiences of women^3^ travelling from outside of CDMX to clinics in CDMX with and without the support of Fondo MARIA. In this paper, we do not take up the question of whether negative feelings dissipate over time as this has been clearly documented in the literature (Broen et al., 2005; Rocca et al., 2015, 2020). Rather, we assess whether accompaniment decreases negative feelings and increases positive feelings more quickly than would be expected without accompaniment at one month and 6 months following an abortion.

## 2 Study context

In Mexico, approximately 50% of unintended pregnancies end in abortion (Guttmacher Institute, 2013). Despite this fact, legal access to abortion in Mexico was highly restricted until recently.^4^ Abortion in Mexico is regulated at the state level. Abortion has been available on request in the first trimester of pregnancy in CDMX since 2007. In most other Mexican states at the time of the study, abortion was only allowed on specific, and limited grounds, and even in cases that met the legal requirements, difficult to access (Grupo de Información en Reproducción Elegida, 2018). Moreover, after the passage of the CDMX abortion law, at least 17 state legislatures in other parts of the country passed increasingly restrictive policies to limit access to abortion services and codify that life begins at conception (Becker & Díaz Olavarrieta, 2013; Beer, 2017). This legal landscape enacted barriers to accessing abortion care among persons living outside of CDMX and allowed authorities to criminalize people seeking abortions. These barriers may have been particularly acute for those living far from CDMX and those with fewer resources from which to draw (Friedman et al., 2019; Saavedra-Avendano et al., 2018; Senderowicz et al., 2018). While norms and beliefs about abortion within Mexico vary state by state and between individuals, research has suggested that the high value placed on motherhood and the influence of the conservative Catholic church within the country create an environment in which abortion is highly stigmatized (Kung et al., 2018; Sorhaindo et al., 2014, 2016). Across Mexico, feminist organizations like Fondo MARIA and feminist activists have mobilized to decriminalize, destigmatize, and provide access to abortion. Since 2018, the “marea verde” [green wave] movement has further catalyzed nationwide action (Felitti & Ramirez Morales, 2020; Álvarez Enríquez, 2020).

## 3 Methods

### 3.1 Data

This analysis draws from an observational longitudinal study that was conducted between January 2017 and June 2018 in Mexico. The study aimed to assess the experiences of women seeking abortions who lived outside of CDMX to understand how Fondo MARIA's accompaniment model influenced abortion stigma, empowerment and autonomy, attitudes and beliefs about reproductive rights, and activism related to reproductive rights. The study recruited three groups of women who lived outside of CDMX who were seeking an abortion: (1) women who travelled to a private or public abortion clinic in CDMX accompanied by Fondo MARIA, (2) women who received support from Fondo MARIA to self-manage their abortion in their state of residence, and (3) women who travelled to private abortion clinics in CDMX without the support of Fondo MARIA. In this paper, we compare the experiences of participants that travelled to CDMX to seek abortion services (Groups 1 and 3 above) because experiences of these groups are most comparable. We refer to the group accompanied by Fondo MARIA as “accompanied” and the group travelling to a clinic on their own as “unaccompanied”, referring only to the accompaniment of Fondo MARIA and not other individuals in participants' social networks who may have provided support throughout the abortion process. Participants were surveyed at three time points: prior to their abortion (referred to as “baseline”), one month after their abortion, and 6 months after their abortion.

Counselors recruited accompanied participants for the study when they first called Fondo MARIA, and prior to receiving any information about the services that Fondo MARIA offered. If interested, the caller provided informed consent and a study staff conducted the baseline survey before reconnecting callers with a counselor to receive accompaniment support. Unaccompanied participants were recruited from two private clinics in CDMX when they arrived at the abortion clinic, prior to counseling. After providing informed consent, unaccompanied participants completed the survey individually on a tablet or with a hard copy, or with an enumerator depending on the participant's preference. Participants were eligible for inclusion in this study if they were 18 years old or older, could provide informed consent, resided outside of CDMX, and were in the first trimester of pregnancy. Accompanied participants sought care at a broader range of clinics than the unaccompanied participants, including both public and private facilities; however, only 8.6% of accompanied participants received care at a public facility (measured at the one-month survey).

Study administrators sent participants links to complete follow-up surveys on Qualtrics at one month after their baseline survey and 6 months after their baseline survey via their preferred follow-up method (text, email, or WhatsApp) and sent up to three reminders to complete each survey. Participants could complete the survey on their own or over the phone. Participants were compensated with $50 pesos (∼$2.65 2017 USD) in cell-phone credit or cash for each survey they completed. Participants were contacted for both follow up surveys regardless of whether they completed the one-month survey. This study was approved by Allendale Investigational Review Board.

### 3.2 Measures

This study sought to understand whether accompaniment was associated with a decrease in negative feelings toward abortion and an increase in positive emotions toward participants’ own abortions over six months.

#### 3.2.1 Dependent variables- emotions

We constructed two indices, one to capture negative and one to capture positive emotions individuals had about their abortion. These indices were based off the method used by Rocca et al. (2020). Participants were asked to indicate whether they had any negative emotions (i.e., guilt, shame, sadness, anguish, nervousness, anxiety, anger, or fear) or positive emotions (i.e., happiness, calmness, decidedness, and relief) about their abortion in the seven previous days. Indices captured the total number of negative (0–8, Chronbach's alpha = .77) or positive emotions (0–4, Chronbach's alpha = .70) at each survey. We also considered each emotion individually to describe the proportion of the sample that felt each emotion. We generated a categorical measure adapting the measure used by Rocca et al. (2020) to capture the combination of emotions that each individual expressed at each time point (categorical characterization of emotions). Participants were categorized as having primarily positive emotions if they expressed half or more of the positive feelings (≥2) and less than half of the negative emotions (<4); primarily negative emotions if they had less than half of the positive emotion (<2) and half or more of the negative emotions (≥4); low emotions if they had less than half of either positive or negative emotions; and, mixed emotions if they had more than half of both positive (≥2) and negative emotions (≥4). We asked an open-ended question that asked participants to describe the principal emotion they had felt in the past seven days about their abortion and recoded responses to into five categories: positive, negative, mixed, neutral, no emotion, and don't know.

#### 3.2.2 Independent variable- accompaniment

Our main predictor of interest was whether the participant was accompanied by Fondo MARIA or unaccompanied in their abortion. For Fondo MARIA, accompaniment is a process that centers each individual's autonomy and agency to make decisions and exercise their own rights. Accompaniment starts when an accompanier and person accompanied come into contact, which can occur at different moments of the abortion seeking process (e.g., seeking information, seeking to schedule an abortion procedure, or seeking post-abortion care). Fondo MARIA' aims to establish equal power between accompaniers and persons accompanied and employs a protocol that responds to diverse needs (individual, familiar, and social) that arise in different moments in seeking abortion care. Fondo MARIA provides emotional support through active listening, engaging with each individual as the expert in their own lives, centering each individual's situation and background, and providing space to talk through conflicting emotions, helping people understand the source of these emotions, and accompanying people through every step of the process (Fondo MARIA, 2015).

#### 3.2.3 Covariates

We considered a range of sociodemographic characteristics and other factors at baseline as potential confounders of the relationship between being accompanied and emotional response to abortion. In terms of sociodemographic variables, we captured age of the participant at baseline, current occupation (not employed or in school, student only, student and employed, and employed only), whether they had any children, whether they had had a previous abortion, their highest level of education (some high school or technical school or less, completed high school or technical school, or some tertiary education or above), and whether they considered themselves religious. We categorized participants as either living above or below the urban-poverty line as defined by El Consejo Nacional de Evaluacion de la Politica de Desarrollo Social by calculating each participants’ maximum per-person household income. (El Consejo Nacional de Evaluación de la Política de Desarrollo Social (CONEVAL), 2019) Variables that characterized the current pregnancy included the gestational age of the pregnancy at time of baseline (1–6 weeks, 7–9 weeks, and 10–12 weeks) and whether the pregnancy happened in the context of a relationship.

We also created an index to measure personal attitudes about abortion by taking the average across the following questions “women that have abortions will be punished by God,” “a woman is to blame for an unwanted pregnancy”, “it's okay that a woman aborts only because she does not want to have a child”, “a woman aborts because she knows what is best for her”, “abortion is a woman's right”, “abortion should be legal”, “abortion should be accessible for everyone” (Chronbach's alpha = .71). These questions were adapted from the Individual Level Abortion Stigma Scale and the Community-Level Abortion Stigma Scale (Cockrill et al., 2013; Sorhaindo et al., 2016). Questions used Likert response options (strongly agree, agree, do not know, disagree, and strongly disagree, coded on a scale of 1–5). Higher scores represented positive attitudes about abortion and negatively phrased questions were reverse coded prior to index creation. Using the same methodology, we created an index representing each participant's level of autonomy at baseline including the questions “I feel in control of my life”, “I feel confident I can overcome difficult situation”, “I have enough time for myself”, “I feel satisfied with my life”, “I believe that every woman has the right to enjoy their sexuality”, and “I can talk openly about my sexuality” (Chronbach's alpha = .75) (Biggs et al., 2017).

We also captured the number of individuals that the participant reported as supporting their abortion process at baseline, from which we generated a dichotomous measure of any support. We also captured whether those who disclosed their abortion to someone at baseline had received a negative reaction.. We measured how participants perceived abortion in their community at baseline using the two separate questions: “The community treats women differently who have had abortions” (Sorhaindo et al., 2016) and “In general, how common do you think abortion is within women in your community”. Separately, we also measured participants' perception of the proportion of their community that believed abortion is a right and abortion should be legal at baseline (Cockrill et al., 2013), combining responses in an index using the methodology described above. We also measured whether participants had engaged in activities to support reproductive rights or abortion in the past month and whether the participant had a conversation(s) in the past month about the right to abortion, creating a dichotomous variable capturing participation in either of these activities at baseline. We constructed a measure of the abortion laws in a participant's state of residence. States with three or fewer exceptions for legal abortion were deemed the “most conservative”, states with four exceptions were “somewhat conservative”, and states with five or more exceptions were considered “least conservative” (Brown et al., 2020). Finally, we captured the distance from CDMX by calculating the drive time from the capital city in each participant's state of residence to CMDX.

### 3.3 Analysis

#### 3.3.1 Descriptive analyses

We summarized all measures of emotional responses to abortion at baseline, one month, and 6 months by accompaniment group. At baseline, we summarized the positive and negative emotion indices by all covariates described above. We tested the statistical significance of these relationships by running bivariable linear regressions at baseline. We also summarize the recategorized open-ended question results and main feelings mentioned at each time point in response to the open-ended question and the categorical characterization of emotions (i.e., primarily positive, primarily negative, low emotions, and mixed emotions) related to the abortion by accompaniment group.

#### 3.3.2 Modeling strategy

We implemented a difference in differences analysis using mixed-effects hierarchical models to assess whether the change in negative or positive emotions from baseline to 6 months after was significantly different among those accompanied compared to those unaccompanied. We used random intercepts to model within-participant correlation. We first fit unadjusted models, including only an interaction between the survey wave (i.e., time) and whether the participant was accompanied. The effect of time was unstructured and was thus allowed to vary. To determine whether baseline differences between the participants who were accompanied and were unaccompanied and potential differential attrition confounded the relationship between accompaniment and emotions, we then ran a model with stabilized inverse probability of treatment and censoring weights (Robins et al., 2000). We opted for weighting techniques to be able to weight both for baseline differences between groups (using stabilized inverse probability of treatment weights) and potential differential attrition based on accompaniment (using stabilized inverse probability of censoring weights).

The propensity score model estimated at baseline to predict accompaniment (the treatment) included the following covariates all measured at baseline: age (continuous), children (yes/no), education (some high school or technical school or less/completed high school or technical school/some tertiary or above), occupation (not student or employed/student only/student and employed/employed only), income under the poverty line (yes/no/don't know), any religious affiliation (yes/no), previous abortion (yes/no), whether the pregnancy happened in the context of a relationship (yes/no), gestational age (<7 weeks, 7–9 weeks, 10–12 weeks), was supported by someone in their abortion (yes/no), personal abortion attitude score (continuous), community attitude score (continuous), perceived commonality of abortion (very common/somewhat common/not at all common), autonomy score (continuous), participation in conversation or activism around abortion and reproductive rights in the past month (yes/no), state abortion legislative context (most conservative state policy contexts/medium conservative state policy contexts/least conservative state policy context), relationship status (single/living together/married/separated, divorced, or widowed), whether the participant perceives the community treats women differently who have had an abortion (agree/disagree or not sure), and any negative reactions to disclosure of abortion seeking (yes/no). We then used this model to create stabilized inverse probability of treatment weights and truncated the weights at the fifth and 95th percentiles (Austin & Stuart, 2015; Cole & Hernán, 2008). We then created stabilized inverse probability of censoring weights to control for the potential that differential attrition between the two groups explained the differences in emotional responses to abortion over time. We created these weights from models of retention which included the same baseline covariates—one model modeled retention at one month and the other at 6 months (See Appendix Table 2 for more information on loss to follow up). We multiplied the inverse probability of treatment weights and the inverse probability of censoring weights to generate a combined weight. We included these weights in the mixed effects difference in differences model described above with the same set of covariates we included in the propensity score model for a doubly robust model and to address slight imbalances in covariate distributions after weighting (See Appendix Table 3). We implemented both effective and size normalization of weights at the participant level in these models. We only present the results using size normalization methodology as results did not differ. Only responses that had a complete set of covariates were included in the main analysis.

We ran several sensitivity analyses. The first involved running an adjusted model using the covariates described above. We also used a multiply imputed dataset (m=25) to replicate our main analysis to address missing outcome and covariate values excluded in the main analysis (described in more detail in the appendix). We also restricted the analyses to those with (non-truncated) propensity scores between 0.5 and 0.95 on the probability scale to address positivity assumptions. Finally, we included only participants with mutual support on the propensity score in another sensitivity analysis. See Appendix.

Analyses were conducted using Stata 15 SE and R Statistical Software.

## 4 Results

### 4.1 Sample characteristics

**Fig. 1:**
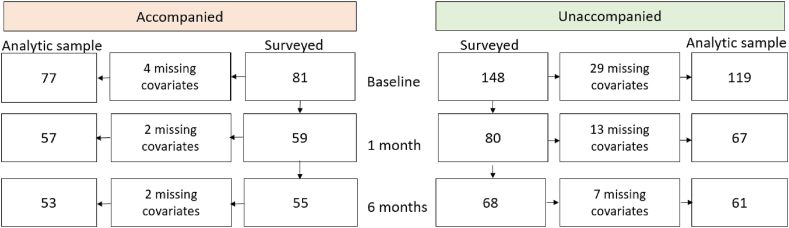
Survey sample flow chart by accompaniment group. Figure shows the number of participants surveyed at each survey over the course of the study by accompaniment group and the total participants included in the analytic sample.

Of the 229 participants that were surveyed at baseline, 196 participants were included in our analysis (85%) based on missing covariate data. By group, 81 accompanied participants and 148 unaccompanied participants completed the baseline survey, 77 (95%) and 119 (80%) of which respectively were included in the analysis (Fig. 1). Forty four percent of the unaccompanied group participated only at baseline and another 38% participated at all three time points. Among the accompanied group, 16% participated only in the baseline survey. Forty eight percent participated at all three time points.

At baseline, over half of the sample was between 18 and 24 years old, had completed some tertiary education or above, and were single (Table 1). Accompanied participants comprised 39% of the sample and unaccompanied participants made up the other 61%. Unaccompanied participants were more likely to report their pregnancy occurred in the context of a relationship, that they were supported by someone at baseline in their abortion, and that they practiced a religion compared to accompanied participants. Specifically, 97% of unaccompanied participants reported feeling supported by at least one person in their abortion process at baseline compared to 86% of accompanied participants. Accompanied participants more often were later in their pregnancy at baseline, had household income under the urban poverty line, were students, reported having had a past abortion, and lived further from CDMX (See Appendix Fig. 1); however, unaccompanied participants were also less likely to report knowing their household income. Those who were accompanied were more likely to have more positive attitudes about abortion at baseline and to have had a conversation or participated in activism around abortion or reproductive rights in the past month compared to unaccompanied participants. Accompanied participants had higher levels of perceived stigma, more often thinking that their community treats women who have abortions differently (66%) than unaccompanied participants (50%). Finally, accompanied participants were more likely to come from both the most conservative state abortion policy contexts and states with the least conservative policy contexts than those who were unaccompanied who were more often from somewhat conservative states.

**Table 1:** Baseline characteristics of study participants travelling to Mexico City for an abortion by accompaniment group, Mexico, 2017–2018

Characteristic	All N (%)	Accompanied %	Unaccompanied %
All	196 (100%)	77 (39.3%)	119 (60.7%)
Age			
18–24	111 (56.6%)	59.7%	54.6%
25–29	45 (23.0%)	19.5%	25.2%
30–34	28 (14.3%)	14.3%	14.3%
35+	12 (6.1%)	6.5%	5.9%
Education			
Some high school or technical school or less	32 (16.3%)	18.2%	15.1%
Completed high school or technical school	62 (31.6%)	29.9%	32.8%
Some tertiary education or above	102 (52.0%)	51.9%	52.1%
Marital status			
Single	126 (64.3%)	64.9%	63.9%
Living together	35 (17.9%)	14.3%	21.0%
Married	21 (10.7%)	10.4%	10.9%
Separated, divorced, widowed	14 (7.1%)	10.4%	5.0%
Pregnancy in the context of a relationship			
No	27 (13.8%)	24.7%	6.7%
Yes	169 (86.2%)	75.3%	93.3%
Occupation			
Not a student or currently working	26 (13.3%)	9.1%	16.0%
Student only	40 (20.4%)	14.3%	24.4%
Student and employed	50 (25.5%)	42.9%	14.3%
Employed only	80 (40.8%)	33.8%	45.4%
Income (Above urban poverty line)			
No	44 (22.4%)	32.5%	16.0%
Yes	125 (63.8%)	59.7%	66.4%
Don't know	27 (13.8%)	7.8%	17.6%
Children			
No	108 (55.1%)	55.8%	54.6%
Yes	88 (44.9%)	44.2%	45.4%
Previous abortion			
No	166 (84.7%)	79.2%	88.2%
Yes	30 (15.3%)	20.8%	11.8%
Practice a religion			
No	48 (24.5%)	35.1%	17.6%
Yes	148 (75.5%)	64.9%	82.4%
Supported by someone in abortion at baseline			
No	15 (7.7%)	14.3%	3.4%
Yes	181 (92.3%)	85.7%	96.6%
Gestational age of pregnancy			
<7 weeks	83 (42.3%)	28.6%	51.3%
7–9 weeks	82 (41.8%)	49.4%	37.0%
10–12 weeks	31 (15.8%)	22.1%	11.8%
Personal attitudes about abortion score^b^			
Quintile 1	62 (31.6%)	16.9%	41.2%
Quintile 2	21 (10.7%)	7.8%	12.6%
Quintile 3	48 (24.5%)	33.8%	18.5%
Quintile 4	40 (20.4%)	26.0%	16.8%
Quintile 5	25 (12.8%)	15.6%	10.9%
Autonomy score^b^			
Quintile 1	53 (27.5%)	33.8%	22.3%
Quintile 2	35 (18.1%)	13.0%	21.6%
Quintile 3	34 (17.6%)	16.9%	18.1%
Quintile 4	37 (19.2%)	16.9%	20.7%
Quintile 5	34 (17.6%)	19.5%	16.4%
Engaged in conversation or activism around right to abortion in the past month			
No	106 (54.1%)	39.0%	63.9%
Yes	90 (45.9%)	61.0%	36.1%
Abortion is common in my community			
Very common	15 (7.7%)	10.4%	5.9%
Somewhat common	88 (44.9%)	46.8%	43.7%
Not common	93 (47.4%)	42.9%	50.4%
Community abortion attitude score^b^^a^			
Quintile 1	45 (23.0%)	27.3%	20.2%
Quintile 2	88 (44.9%)	42.9%	46.2%
Quintile 4	45 (23.0%)	24.7%	21.8%
Quintile 5	18 (9.2%)	5.2%	11.8%
Perceived stigma- Community treats someone differently who has had an abortion			
No	85 (43.6%)	33.8%	50.0%
Yes	110 (56.4%)	66.2%	50.0%
Negative reaction after telling someone about abortion			
No	152 (77.6%)	79.2%	76.5%
Yes	44 (22.4%)	20.8%	23.5%
State abortion policy context			
2-3 legal exceptions	34 (17.3%)	26.0%	11.8%
4 legal exceptions	131 (66.8%)	50.6%	77.3%
5 or more legal exceptions	31 (15.8%)	23.4%	10.9%
State distance from Mexico City (CDMX)			
<2 h	100 (52.3%)	29.9%	65.3%
2–4 h	40 (20.5%)	29.9%	14.4%
5+ hours	55 (28.2%)	40.3%	20.3%

a No one in quintile 3.

b Higher scores represent more supportive attitudes, more autonomy, and more supportive community attitudes.

### 4.2 Emotions related to the abortion at baseline

**Fig. 2:**
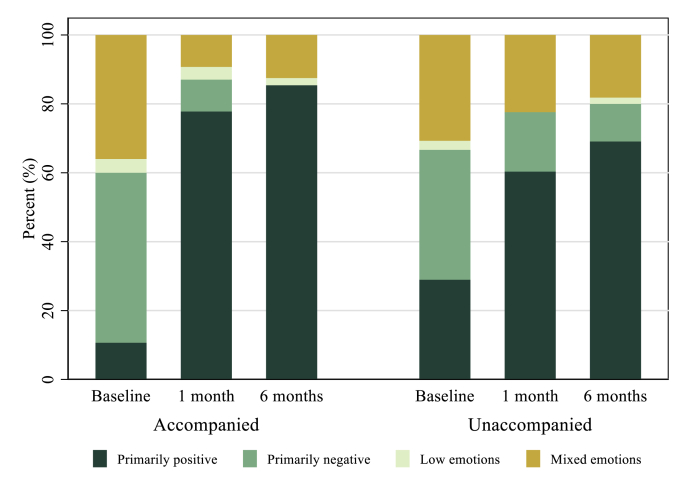
Abortion emotion profiles six months following abortion among study participants travelling to Mexico City for an abortion by accompaniment group, Mexico, 2017–2018. Participants were categorized as having primarily positive emotions if they expressed half or more of the positive feelings (≥2) and less than half of the negative emotions (<4); primarily negative emotions if they had less than half of the positive emotions (<2) and half or more of the negative emotions (≥4); low emotions if they had less than half of either positive or negative emotions; and, mixed emotions if they had more than half of both positive (≥2) and negative emotions (≥4). Figure shows descriptive, unadjusted results. The number of participants represented at each time point were as follows: accompanied baseline n = 75, accompanied 1 month n = 54, accompanied 6 months n = 48; unaccompanied baseline n = 114, unaccompanied 1 month = 58, unaccompanied 6 months n = 55.

At baseline, before they had obtained an abortion, participants in the full sample on average had 4.6 of 8 negative emotions and 1.8 of 4 positive emotions. At baseline, unaccompanied participants had fewer negative emotions (4.4 vs. 4.9) and more positive emotions (1.9 vs. 1.7); these differences were not statistically significantly different, although the negative emotions approached conventional statistical significance (two-sided *t*-test, p = .06) (Table 2). At baseline, the most prevalent negative emotions included anguish, nervousness, anxiety, fear, and sadness. Considering positive emotions, almost all participants felt decided about going through their abortion process at baseline, but a minority felt happy, calm, or relieved. Examining the categorical characterization of feelings, a higher proportion of accompanied participants felt primarily negative (49% vs. 38%), a similar proportion felt mixed (36% vs. 30%) and a lesser proportion felt primarily positive (11% vs. 29%) compared to unaccompanied participants (Fig. 2). Between 3 and 4% in both groups had low emotion (i.e., less than half of either positive or negative emotions) about their abortion at baseline. In open responses, most participants highlighted negative emotions as the primary feeling they felt about their abortion (88% in the accompanied group and 75% in the unaccompanied group, data not shown) at baseline. The most common feelings named in open-ended questions were sadness, nervousness, feeling scared, and feeling guilty.

**Table 2:** Emotion indices and individual feelings over six months following an abortion among women who travelled to Mexico City by accompaniment group (n_obs_ = 417, n_par_ = 196), Mexico, 2017–2018

	Accompanied				Unaccompanied			
	Baseline (n = 76)	1 month (n = 55)	6 months (n = 50)	% change from baseline to 6 months	Baseline (n = 118)	1 month (n = 60)	6 months (n = 58)	% change from baseline to 6 months
Negative emotions								
*Index*	*4.89*	*1.98*	*1.66*	−66.1%	*4.42*	*2.66*	*2.20*	*−50.2%*
Anguish	92.2%	19.6%	13.7%	−85.1%	76.5%	31.7%	19.6%	*−74.4%*
Nervous	92.2%	20.0%	27.5%	−70.2%	89.7%	31.7%	22.4%	*−75.0%*
Scared	87.0%	21.4%	27.5%	−68.4%	80.7%	30.0%	23.2%	*−71.3%*
Anxious	83.1%	28.6%	26.0%	−68.7%	70.6%	34.4%	25.9%	*−63.3%*
Sad	75.3%	47.3%	35.3%	−53.1%	72.3%	60.0%	43.1%	*−40.4%*
Guilt	57.1%	35.7%	27.5%	−51.8%	54.2%	42.4%	51.7%	*−4.6%*
Angry	51.9%	20.0%	19.6%	−62.2%	47.1%	46.7%	35.1%	*−25.5%*
Shame	39.5%	25.0%	17.6%	−55.4%	33.1%	16.7%	29.3%	*−11.5%*
Positive emotions								
*Index*	*1.72*	*3.22*	*3.45*	100.6%	*1.87*	*2.98*	*3.03*	*62.0%*
Decided	93.4%	87.3%	88.0%	−5.8%	86.4%	83.3%	82.8%	*−4.2%*
Relieved	32.9%	87.5%	92.2%	180.2%	39.8%	83.6%	81.0%	*103.5%*
Calm	31.2%	87.5%	92.0%	194.9%	45.4%	79.0%	78.0%	*71.8%*
Happy	15.6%	60.1%	68.6%	339.7%	15.4%	54.1%	62.1%	*303.2%*

Note that n_obs_ represents the number of person-time observations and n_par_ represents the total number of participants; 2 participants had missing values for the outcome at baseline but were included in later time points.

Participants in the sample had fewer negative feelings and more positive feelings at baseline if they got pregnant in the context of a relationship (p = .01), had higher levels of autonomy (p < .001), and perceived their community to be more supportive of abortion (p = .02) (Appendix Table 1). Participants had more negative feelings at baseline if they had children (p = .01) or perceived abortion stigma in their communities (p = .04).

### 4.3 Emotions related to the abortion over time

#### 4.3.1 Descriptive results

Over time, negative feelings decreased and positive feelings increased (Table 2). These changes were more pronounced in the accompanied group than the unaccompanied group. In the accompanied group, participants reported having on average 2.0 negative feelings at one month and 1.7 negative feelings at 6 months, a 60% and 66% decrease from baseline respectively. Among the unaccompanied group, despite having lower negative emotions at baseline, negative emotions decreased to a lesser extent than the accompanied group. Unaccompanied participants had on average 2.7 and 2.2 negative emotions at one and 6 months, a decrease of 39% and 50% respectively. Among the individual negative emotions measured, the biggest decreases were observed for anguish, nervousness, and fear. Among the unaccompanied group, feelings of shame and guilt changed little from baseline; over 50% of participants at baseline and 6 months reported feeling guilty. In the accompanied group, the levels of guilt decreased from 57% at baseline to 28% at 6 months. For feelings of shame, the proportion of accompanied participants reporting shame decreased from 40% at baseline to 18% at 6 months compared to a change of 33% to 29% from baseline to 6 months among the unaccompanied group.

Positive feelings also increased to a greater extent in the accompanied group, increasing to an average of 3.2 and 3.5 positive feelings at one and 6 months compared to 3.0 at both times points among the unaccompanied group. Almost all accompanied participants reported feeling calm and relieved at 6 months (92%). Among the unaccompanied group, these feelings also increased, but to a lesser extent, so that by 6 months 78% felt calm and 81% felt relieved. In both groups, feelings of happiness were least prevalent at 6 months. The most common feelings mentioned in the open-ended questions at one and 6 months included relief, tranquility, sadness, and guilt.

Examining the categorical measure of emotions, at one month 78% and 60% of accompanied and unaccompanied participants respectively felt primarily positive (Fig. 2), an inversion of the pattern observed at baseline. A smaller proportion of accompanied participants felt mixed emotions (9%) or primarily negative emotions (9%) than the unaccompanied group (23% and 18% respectively). At 6 months, 85% of accompanied participants felt primarily positive compared to 69% of unaccompanied participants. Coding of the open-ended questions produced similar results; however, more participants reported not knowing what their primary emotion was at one and 6 months. At 6 months, the unaccompanied group named negative emotions in the open-ended question to a greater extent than the accompanied group (33% vs. 19% in the accompanied group).

#### 4.3.2 Model results

**Fig. 3:**
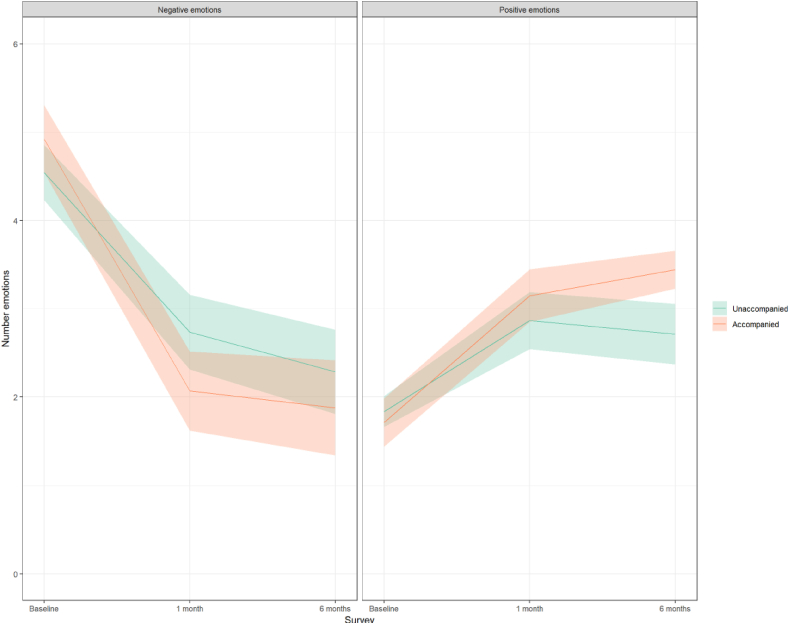
Predicted negative and positive emotion indices from doubly robust inverse probability weighted models by accompaniment group six months after an abortion, Mexico, 2017–2018. Mean predicted values using doubly robust inverse probability weighted models. Note that the confidence intervals test the differences between each group at the given time point, while the interaction in the model tests the difference between the change from baseline between the accompanied and unaccompanied groups.

Participants who were accompanied had larger and statistically significant decreases in negative emotions and larger increases in positive emotions at 6 months compared to those that were unaccompanied after their abortion (Table 3); however, the difference in positive emotions from baseline was not statistically significantly different between accompanied and unaccompanied participants at one month. Confidence intervals at this time point, however, barely included values under 0. Considering negative emotions, our model predicted that those who were accompanied had a decrease of 0.8 more negative emotions six months after their abortion than unaccompanied participants did (95% CI: 1.54 to −0.03). Among unaccompanied participants, negative emotions decreased significantly from baseline, but to a lesser extent than accompanied participants did. Among unaccompanied participants, the number of negative emotions decreased by 1.8 and 2.3 from baseline to one and 6 months respectively (Fig. 3). The number of negative emotions experienced by accompanied participants about their abortion decreased by 2.9 and 3.0 from baseline at one and 6 months. Examining positive feelings, unaccompanied participants had increases of about one positive feeling from baseline to both one and 6 months. At six months, positive feelings increased on average by 0.85 feelings more from baseline among accompanied participants compared to unaccompanied participants (95% CI: 0.33–1.37), translating to a difference of 1.7 more positive feelings than baseline among the accompanied group.

**Table 3:** Model results examining changes in negative and positive emotion indices six months following an abortion among women who travelled to Mexico City by accompaniment group, Mexico, 2017–2018

	Negative feelings index		Positive feelings index	
	Unadjusted (n_obs_ = 413, n_part_ = 196)	Doubly robust (n_obs_ = 408, n_part_ = 193)	Unadjusted (n_obs_ = 413, n_part_ = 194)	Doubly robust (n_obs_ = 409, n_part_ = 191)
Accompanied	0.47 (−0.06–1.00)	0.38 (−0.15–0.91)	−0.14 (−0.46–0.18)	−0.12 (−0.45–0.21)
1 month	−1.75*** (−2.18 to −1.31)	−1.81*** (−2.24 to −1.37)	1.06*** (0.77–1.34)	1.03*** (0.64–1.42)
6 months	−2.28*** (−2.73 to −1.83)	−2.25*** (−2.74 to −1.77)	1.11*** (0.81–1.40)	0.87*** (0.47–1.28)
1 month * Accompanied	−1.05** (−1.69 to −0.41)	−1.05** (−1.69 to −0.40)	0.40 (−0.02–0.82)	0.40 (−0.12–0.93)
6 months * Accompanied	−0.82* (−1.48 to −0.16)	−0.79* (−1.54 to −0.03)	0.58** (0.14–1.01)	0.85** (0.33–1.37)

*p < .05 **p < .01 ***p < .001.

Table shows regression coefficients and 95% confidence intervals. Doubly robust generalized linear models (gaussian distribution and identity link) include inverse probability weights and covariate adjustment, n_obs_ represents all observations across all time points included in the model n_part_ represents the number of participants included in the model. The covariates included are: age, children, education, occupation, income under the poverty line, any religious affiliation, previous abortion, whether the pregnancy happened in the context of a partnership, gestational age, was supported by someone in their abortion, personal abortion attitude score, community attitude score, perceived commonality of abortion, autonomy score, participation in conversation or activism around abortion and reproductive rights in the past month, state abortion legislative context, relationship status, whether the participant perceives the community treats women differently who have had an abortion, and any negative reactions to disclosure of abortion seeking.

Sensitivity analyses broadly did not change the conclusions of the analysis, particularly for negative emotions (See Appendix Table 4); however, the model including only observations with mutual support in propensity scores resulted in the coefficient on the interaction of accompaniment and 6 months to no longer be significant (although 1 month was) and positive emotion results were sensitive to using imputed datasets.

## 5 Discussion

We explored whether feminist accompaniment from Fondo MARIA was associated with decreased negative feelings and increased positive emotions that women seeking abortion services in CDMX felt about their abortion 6 months after their abortion compared women who were unaccompanied. Our results found that women accompanied by Fondo MARIA had larger decreases in negative feelings and larger increases in positive feelings toward their abortion over time compared to those who were not accompanied; however, results for positive emotions were more sensitive and did not hold when using imputed datasets. By endline, the majority of accompanied respondents had primarily positive feelings about their abortion. This meant that while at baseline feelings were comparable between those accompanied and unaccompanied, by 6 months after the abortion, the proportion of participants who reported feeling guilty, angry, and shameful had decreased to a greater extent among those accompanied then those who were unaccompanied. Similarly, the proportion of participants reporting being calm and relieved increased to a greater extent among accompanied participants than unaccompanied participants. As in other studies, the vast majority of participants reported being decided about their abortion at all time points and negative emotions decreased after an abortion (Rocca et al., 2020).

This study drew on observational data to assess how accompaniment influenced emotions women had about their abortions and suggests that accompaniment may contribute to a more positive frame of the abortion experience. While the results cannot speak to the exact mechanisms through which accompaniment acts on emotional responses to abortion, research on interventions that provide support to people after their abortion provides insight. In the US, a study introducing patients to a “culture of support” in the form of providing evidence-based information, validating messages, and information about groups and services that support people in exercising their reproductive autonomy, found that the intervention helped participants reject negative judgmental attitudes (Littman et al., 2009). A mixed-methods evaluation of story circles run by feminist activists in Mexico that provided space for women to come together after their abortion to share their stories, create connections with each other, and explore what their decision has meant for their lives found that the circles quantitatively decreased feelings of isolation and qualitatively helped reduce stigmatizing feelings. They found that the circles empowered individuals to reframe abortion as a positive and life-affirming decision from a negative and stigmatizing one (Belfrage et al., 2019). People involved in abortion care and support in other countries can draw on this work as the legal landscape becomes more restrictive; this is particularly true in the United States, where people in nearly half of all states are expected to have to travel across state lines for abortion services following the gutting of constitutional protections for abortion access.

As the accompaniment model centers people's lives and goals, validates and affirms their choices, normalizes and emphasizes the right to abortion, and provides social support, it has the potential to create new normative contexts and social relations around abortion that may be transformational. The emphasis on each person's goals and their right to self-determination and autonomy may enable people seeking abortion to view their decision as one that is valid and legitimate, and resist the predominant narratives framing abortion as something that is transgressive and morally wrong (Kumar et al., 2009). This framing could explicitly invert and resist abortion stigma by allowing people seeking abortion to put their own autonomy and goals first, instead of centering the socially ascribed roles of mother, caregiver, and procreator (Kumar et al., 2009; Olivia, 2011). Accompaniment facilitates conversations that affirm the decisions of the person seeking abortion care and helps situate the feelings and experiences of the individual in a larger community of people seeking abortions, potentially helping the person feel less isolated in their experience and providing social support that may be lacking in their own social network. In providing spaces for conversation, accompaniers help people seeking abortion to explore and reflect on any negative feelings they might have, such as guilt or shame, and respond in solidarity, contributing to challenging and dislodging societal expectations of what people seeking abortions are supposed to feel. This may include helping people identify ways their faith or religion supports abortion care. While clinic-based models often have a rights-based orientation to their counseling, the feminist orientation of accompaniment models that go beyond ensuring access to safe abortion services, may shift the hierarchical nature of clinic-based care and allow for the reframing of abortion as something from which individuals can draw power (Erdman et al., 2018; Veldhuis et al., 2022). Accompaniment also supports someone seeking abortion care from the beginning process to after their procedure, while clinics often can only provide support to the patient while at the site. Future work could help understand the precise elements of the accompaniment process that may alleviate negative feelings and promote positive ones and explore how other accompaniment organizations, clinics, and support people could incorporate principles of the feminist accompaniment model when supporting people who are seeking or have obtained an abortion. Given the persistence of some stigma-related negative feelings such as guilt and shame in both groups, additional work could seek to understand whether additional interventions or approaches could further support people to dislodge abortion-related stigma.

There are various limitations to our study. While we employed multiple analyses to rule out differences between the accompanied and unaccompanied groups and attrition as the cause of the differences in emotions over time, determinants of emotions (and potentially loss to follow up) are complex and multifaceted; there are factors that we could not control for in our analyses that limit the conclusions that can be drawn. First, we did not measure some variables such as mental health prior to seeking abortion, self-efficacy, or resiliency, or other sources of accompaniment among those not accompanied by Fondo MARIA, all factors that might contribute or predict emotional responses after abortion (Robinson et al., 2009). We did, however, control for the majority of factors that had been identified as potentially influential in emotional responses after an abortion, such as support from a participant's social network, religiosity, perceived stigma, and measures of the broader socio-cultural context (Littman et al., 2009). The inclusion of random effects also helps adjust for time invariant factors. Second, we could not control for how clinic-related factors influenced emotions held by participants about their abortion. Third, the timing of the baseline survey varied between accompanied and unaccompanied participants. While accompanied participants may have reported more negative feelings at baseline compared to unaccompanied participants because they were surveyed earlier in their abortion seeking process, differences in levels and prevalence of emotions at one month and 6 months suggests more positive (and less negative) emotions among the accompanied group at endline, independent of levels at baseline. Lastly, the sample of participants included is also relatively small and not representative of all abortion seekers in Mexico City—the sample was more highly educated than previous research examining abortion seekers travelling from outside CMDX at public facilities has found, likely because those who are able to travel further distances for abortion services have more resources (Garnsey et al., 2022; Jacobson et al., 2022; Senderowicz et al., 2018).

## 6 Conclusion

This paper suggests that accompaniment may help increase positive emotions and decrease negative emotions associated with abortion among people seeking abortion, especially those related to abortion stigma. Through centering each individual and transforming normative contexts around abortion, accompaniment models such as Fondo MARIA, may be actively destigmatizing abortion, promoting the agency and autonomy of all people, and providing needed social support.

## Funding

This work was supported by an anonymous foundation donor. The donor had no part in the study design, collection, analysis, interpretation of the data, writing the paper, or the decision to submit the article for publication.

## Author statement

Alexandra Wollum: Methodology, Formal Analysis, Writing-Original Draft Sofía Garduño Huerta: Conceptualization, Resources, Project administration, Writing-Review and Editing Oriana López Uribe: Conceptualization, Project administration, Writing-Review and Editing Camille Garnsey: Data curation, Writing-Review & Editing S. Michael Gaddis: Methodology, Writing-Review & Editing Sarah E. Baum: Conceptualization, Methodology, Funding acquisition, Writing-Review and Editing, Supervision Brianna Keefe-Oates: Conceptualization, Methodology, Writing-Review and Editing, Supervision, Project administration.

## Ethical statement

This study was approved by Allendale Investigational Review Board [Study: IBISCA 102016] and the research was conducted in accordance with national and local guidelines and regulatory procedures in Mexico. We did not seek ethical approval from a Mexican IRB due to concerns about potential negative implications for Fondo MARIA's work outside of CDMX.

## Declarations of competing interest

None.

## Data Availability

The data that have been used are confidential.

## Footnotes

1 Fondo MARIA uses the word “women” to describe their work, while recognizing that not just those who identify as cisgender women seek or need abortions.

2 At the time of the study, abortion on broad grounds was only legal in Mexico City.

3 We recognize that not all people who seek and receive abortion services identify as women and yet much of the existing literature on this topic specifically refers to women. At the time we conducted the survey, we used the word ‘women’ in our recruitment process. To be both accurate and inclusive, we use the terms ‘woman/women’ and the prounouns ‘she/her’ when referring to our own data and citing studies that specified recruiting women. We use the more gender-inclusive term ‘people’ in all other instances, as people of all genders seek abortion care and may benefit from abortion accompaniment.

4 Starting in 2019 a number of states expanded access to abortion in most circumstances (Oaxaca, Hidalgo, Veracruz, Baja California, Colima, and Sinaloa) and in September 2021, the Supreme Court of Mexico voted to not criminalize abortion (Kitroeff & Lopez, 2021; “Mexico's Oaxaca state legalizes abortion in historic move for Catholic nation,” 2019; Solomon, 2021a, 2021b). However, abortion is regulated at the state-level in Mexico, and substantial obstacles to implementation remain. This study took place before these laws were passed.
