# Supplementary material for: The influence of feminist abortion accompaniment on emotions related to abortion: A longitudinal observational study in Mexico
Source: SSM Popul Health. 2022 Oct 4;19:101259. doi: 10.1016/j.ssmph.2022.101259 (PMC9552094; doi:10.1016/j.ssmph.2022.101259)
Supplement: Multimedia component 1 [file mmc1.docx]

***Appendix***
Figure 1: State of residence for accompanied and unaccompanied study participants (n=194)


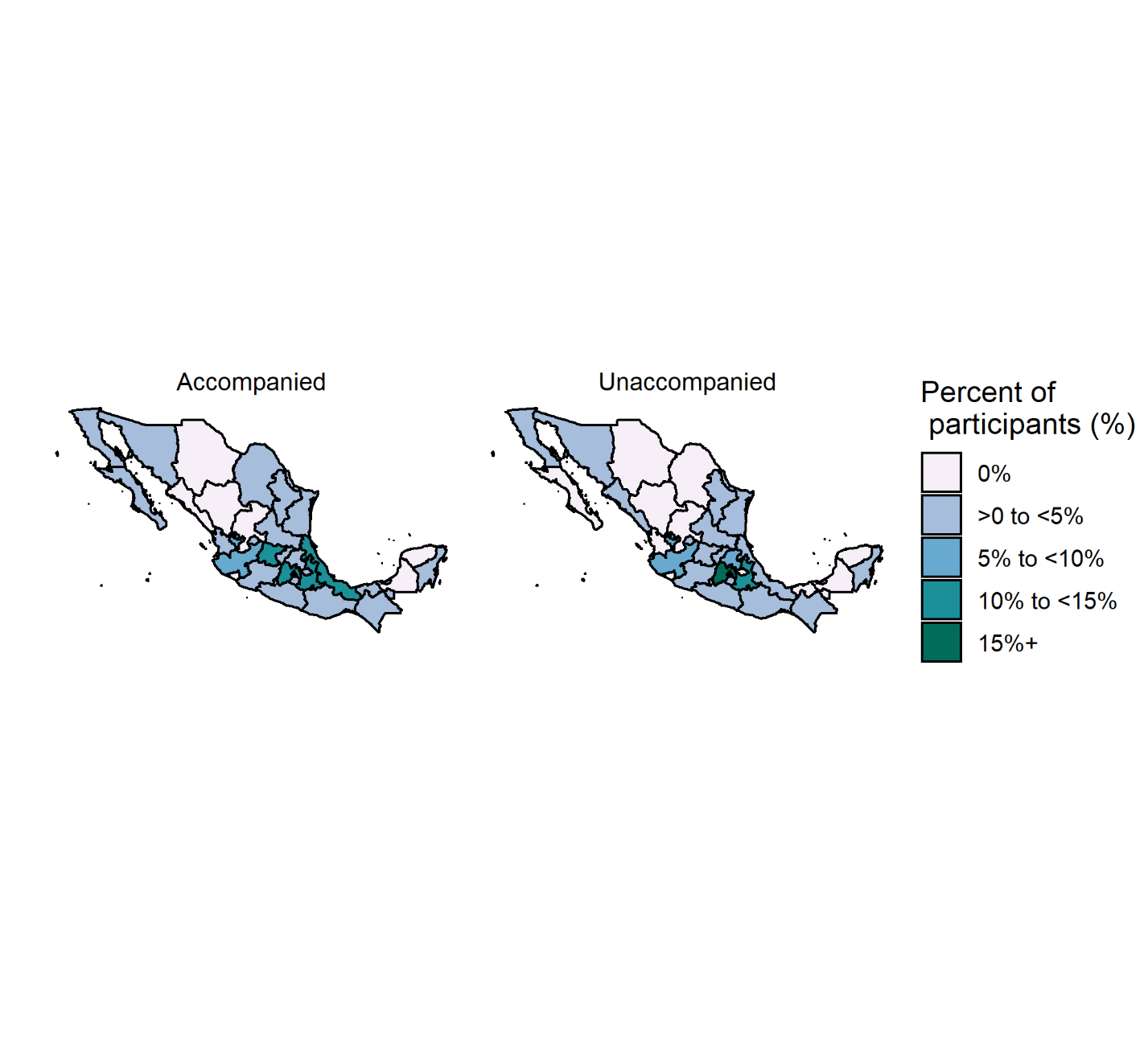


Table 1: Average negative and positive emotions by baseline characteristics among women who travelled to Mexico City, Mexico, 2017-2018

| Characteristic | Mean negative emotion index  (range: 0-8) | Mean positive emotion index  (range: 0-4) |
| --- | --- | --- |
| All | 4.60 | 1.81 |
| Age |  |  |
| 18-24 | 4.54 | 1.86 |
| 25-29 | 4.38 | 1.93 |
| 30-34 | 5.21 | 1.41 |
| 35+ | 4.67 | 1.67 |
| Education |  |  |
| Some high school or technical school or less | 5.09 | 1.90 |
| Completed high school or technical school | 4.48 | 1.92 |
| Some tertiary education or above | 4.53 | 1.70 |
| Marital status |  |  |
| Single | 4.57 | 1.82 |
| Living together | 4.29 | 1.97 |
| Married | 4.57 | 1.90 |
| Separated, divorced, widowed | 5.79 | 1.07 |
| Pregnancy in the context of a relationship |  |  |
| No | 5.37 | 1.37 |
| Yes | 4.49 | 1.87 |
| Occupation |  |  |
| Not a student or currently working | 4.69 | 1.92 |
| Student only | 4.28 | 1.72 |
| Student and employed | 4.51 | 1.88 |
| Employed only | 4.80 | 1.75 |
| Income (Above urban poverty line) |  |  |
| No | 4.47 | 1.86 |
| Yes | 4.58 | 1.83 |
| Don’t know | 4.96 | 1.63 |
| Children |  |  |
| No | 4.32 | 1.84 |
| Yes | 4.95 | 1.76 |
| Previous abortion |  |  |
| No | 4.60 | 1.76 |
| Yes | 4.67 | 2.07 |
| Practice a religion |  |  |
| No | 4.60 | 1.64 |
| Yes | 4.61 | 1.85 |
| Supported by someone in abortion at baseline |  |  |
| No | 4.80 | 1.67 |
| Yes | 4.59 | 1.81 |
| Gestational age of pregnancy |  |  |
| <7 weeks | 4.49 | 1.82 |
| 7-9 weeks | 4.72 | 1.75 |
| 10-12 weeks | 4.57 | 1.93 |
| Personal attitudes about abortion score^ |  |  |
| Quintile 1 | 5.06 | 1.64 |
| Quintile 2 | 4.48 | 1.90 |
| Quintile 3 | 4.28 | 1.78 |
| Quintile 4 | 4.28 | 2.05 |
| Quintile 5 | 4.75 | 1.76 |
| Autonomy score^ |  |  |
| Quintile 1 | 5.25 | 1.27 |
| Quintile 2 | 4.89 | 1.94 |
| Quintile 3 | 4.56 | 1.73 |
| Quintile 4 | 4.57 | 1.75 |
| Quintile 5 | 3.24 | 2.70 |
| Engaged in conversation or activism around right to abortion in the past month |  |  |
| No | 4.43 | 1.80 |
| Yes | 4.82 | 1.80 |
| Abortion is common in my community |  |  |
| Very common | 4.50 | 1.47 |
| Somewhat common | 4.49 | 1.94 |
| Not common | 4.74 | 1.73 |
| Community abortion attitude score^* |  |  |
| Quintile 1 | 4.91 | 1.57 |
| Quintile 2 | 4.78 | 1.79 |
| Quintile 4 | 4.58 | 1.86 |
| Quintile 5 | 3.00 | 2.28 |
| Perceived stigma- Community treats someone differently who has had an abortion |  |  |
| No | 4.31 | 1.90 |
| Yes | 4.82 | 1.72 |
| Negative reaction after telling someone about abortion |  |  |
| No | 4.54 | 1.85 |
| Yes | 4.84 | 1.63 |
| State abortion policy context |  |  |
| 2-3 legal exceptions | 4.85 | 1.65 |
| 4 legal exceptions | 4.59 | 1.80 |
| 5 or more legal exceptions | 4.42 | 1.97 |

*No one in quintile 3

^higher scores represent more supportive attitudes, more autonomy, and more supportive community attitudes

Table 2: Baseline emotion indices and covariates comparing those retained in the study versus those lost to follow up by accompaniment group

| **Variable** | **Accompanied** | | **Unaccompanied** | |
| --- | --- | --- | --- | --- |
|  | Retained  (n=65) | Lost to follow up  (n=12) | Retained  (n=67) | Lost to follow up  (n=52) |
| Negative emotions index | 4.8 | 5.3 | 4.6 | 4.2 |
| Positive emotions index | 1.8 | 1.3 | 2.0 | 1.7 |
| Age | 24.7 | 26.0 | 24.8 | 25.9 |
| Has children | 40.0% | 66.7% | 41.8% | 50.0% |
| Education |  |  |  |  |
| Some high school or technical school or less | 13.9% | 41.7% | 13.4% | 17.3% |
| Completed high school or technical school | 29.2% | 33.3% | 31.3% | 34.6% |
| Some tertiary or above | 56.9% | 25.0% | 55.2% | 48.1% |
| Occupation |  |  |  |  |
| Not student or employed | 9.2% | 8.3% | 13.4% | 19.2% |
| Student only | 15.4% | 8.3% | 23.9% | 25.0% |
| Student and employed | 46.2% | 25.0% | 13.4% | 15.4% |
| Employed only | 29.2% | 58.3% | 49.3% | 40.4% |
| Religiously affiliated | 63.1% | 75.0% | 80.6% | 84.6% |
| Above urban poverty line |  |  |  |  |
| No | 29.2% | 50.0% | 17.9% | 13.5% |
| Yes | 63.1% | 41.7% | 61.2% | 73.1% |
| Don’t know | 7.7% | 8.3% | 20.9% | 13.5% |
| Pregnancy in context of relationship | 78.5% | 58.3% | 94.0% | 92.3% |
| Gestational age |  |  |  |  |
| <7 weeks | 29.2% | 25.0% | 55.2% | 46.2% |
| 7-9 weeks | 47.7% | 58.3% | 34.3% | 40.4% |
| 10-12 weeks | 23.1% | 16.7% | 10.5% | 13.5% |
| Supported in abortion at baseline | 86.2% | 83.3% | 100% | 92.3% |
| Personal abortion attitudes score | 4.34 | 4.19 | 4.15 | 3.93 |
| Autonomy score | 3.89 | 3.51 | 3.97 | 3.83 |
| Conversation or activism around abortion/reproductive rights in last month | 64.6% | 41.7% | 44.8% | 25.0% |
| Community attitude score | 2.07 | 2.29 | 2.31 | 2.20 |
| Abortion is common in the community |  |  |  |  |
| Very common | 9.2% | 16.7% | 6.0% | 5.8% |
| Somewhat common | 49.2% | 33.3% | 41.8% | 46.2% |
| Not common | 41.5 | 50.0% | 52.2% | 48.1% |
| State abortion context |  |  |  |  |
| 2-3 legal exceptions | 26.2% | 25.0% | 9.0% | 15.4% |
| 4 legal exceptions | 47.7% | 66.7% | 80.6% | 73.1% |
| 5 or more legal exceptions | 26.2% | 8.3% | 10.5% | 11.5% |
| Relationship status |  |  |  |  |
| Single | 67.7% | 50.0% | 71.6% | 53.9% |
| Living together | 15.4% | 8.3% | 14.9% | 26.9% |
| Married | 7.7% | 25.0% | 7.5% | 15.4% |
| Separated, divorced, or widowed | 9.2% | 16.7% | 6.0% | 3.9% |
| Community treats someone differently who has had an abortion | 69.2% | 50.0% | 57.6% | 40.4% |
| Previous abortion | 20.0% | 25.0% | 14.9% | 7.7% |
| Negative reaction after telling someone about abortion | 23.1% | 8.3% | 20.9% | 26.9% |

Those retained are those that responded to at least two surveys (baseline and either/and 1 month and six months).

Table 3: Weighted means and proportions of baseline covariates using inverse probability treatment weight by accompaniment group

| **Variable** | **Accompanied** | **Unaccompanied** |
| --- | --- | --- |
| Age | 25.03 | 25.28 |
| Has children | 43.05% | 44.14% |
| Education |  |  |
| Some high school or technical school or less | 17.92% | 17.20% |
| Completed high school or technical school | 26.59% | 32.89% |
| Some tertiary or above | 55.49% | 49.91% |
| Occupation |  |  |
| Not student or employed | 11.19% | 14.17% |
| Student only | 16.10% | 19.86% |
| Student and employed | 33.45% | 24.46% |
| Employed only | 39.26% | 41.50% |
| Religiously affiliated | 71.66% | 78.31% |
| Above urban poverty line |  |  |
| No | 27.21% | 18.33% |
| Yes | 59.89% | 62.89% |
| Don’t know | 12.90% | 18.78% |
| Pregnancy in context of partnership | 82.43% | 88.46% |
| Gestational age |  |  |
| <7 weeks | 34.21% | 43.72% |
| 7-9 weeks | 43.08% | 38.78% |
| 10-12 weeks | 22.71% | 17.50% |
| Supported in abortion at baseline | 91.25% | 96.33% |
| Personal abortion attitudes score | 4.27 | 4.10 |
| Autonomy score | 3.85 | 3.86 |
| Conversation or activism around abortion/reproductive rights in last month | 55.16% | 42.81% |
| Community attitude score | 2.19 | 2.25 |
| Abortion is common in the community |  |  |
| Very common | 12.47% | 7.14% |
| Somewhat common | 46.22% | 44.21% |
| Not common | 41.30% | 48.65% |
| State abortion context |  |  |
| 2-3 legal exceptions | 21.27% | 17.85% |
| 4 legal exceptions | 61.26% | 70.21% |
| 5 or more legal exceptions | 17.46% | 11.93% |
| Relationship status |  |  |
| Single | 67.74% | 64.79% |
| Living together | 14.55% | 18.44% |
| Married | 11.08% | 11.71% |
| Separated, divorced, or widowed | 6.63% | 5.06% |
| Community treats someone differently who has had an abortion | 54.35% | 51.32% |
| Previous abortion | 15.81% | 16.06% |
| Negative reaction after telling someone about abortion | 18.63% | 22.34% |

*Imputation*

Missing variables included in the analysis were imputed using Amelia software in R. For variables that were generated from a set of variables (i.e., negative emotion index, positive emotion index, community attitude score, autonomy score, personal abortion attitude score, engaged in conversation or activism around right to abortion in the past month, occupation), we imputed all subcomponents and calculated the variable after imputation was complete. The following variables were given as nominal variables within Amelia: all emotion variables, activities to support reproductive rights or abortion in the past month, conversation(s) in the past month about the right to abortion, negative reactions to disclosure of abortion, student status, employment status, previous abortion, religious affiliation, relationship status, below the urban-poverty line, highest level of education, pregnancy in the context of a partnership, gestational age at baseline, support from someone in the abortion process, had children, and perceived commonality of abortion in the community. We allowed for squared terms in the effect of time. For Likert variables, we enforced bounds of 1 and 5 within the imputation. We estimated inverse probability weights for each imputation. We ran unadjusted, adjusted, and inverse probability weighted models as described in the main analysis using the MI functions in Stata to combine results across the 25 imputations.

Table 4: Model results examining changes in negative and positive emotion indices six months following an abortion among women who travelled to Mexico City by accompaniment group for all sensitivity analyses

|  | Negative emotions | | | | | | Positive emotions | | | | | |
| --- | --- | --- | --- | --- | --- | --- | --- | --- | --- | --- | --- | --- |
|  | Adjusted  (n_obs_=408) | Imputed unadjusted  ( n_obs_=491) | Imputed adjusted  (n_obs_=491) | Imputed IPW  ( n_obs_=491) | Restricted propensity score (.05 - .95)  (n_obs_=302) | Mutual support  (n_obs_=268) | Adjusted  (n_obs_=409) | Imputed unadjusted  (n_obs_ =491) | Imputed adjusted  (n_obs_ =491) | Imputed IPW  (n_obs_ =491) | Restricted propensity score (.05 - .95)  (n_obs_ =301) | Mutual support  (n_obs_ =267) |
| Accompanied | 0.27  (-0.31 - 0.86) | .55*  (.05 - 1.05) | .26  (-.29 - .80) | .31  (-.15 -.78) | .25  (-.26 - .76) | .55*  (.01 - 1.08) | -0.03  (-0.39 - 0.32) | -.10  (-.40 - .21) | -.02  (-.35 - .32) | .002  (-.33 - .33) | -.06  (-.39 - 0.27) | -.24  (-.61 - .13) |
| 1 month | -1.70***  (-2.13 - -1.26) | -1.83***  (-2.25 - -1.42) | -1.82***  (-2.22-1.41) | -1.90  (-2.35 -1.45) | -1.77***  (-2.25 - -1.28) | -1.87***  (-2.34- -1.40) | 0.99***  (0.71 - 1.29) | 1.06***  (.80 - 1.33) | 1.05***  (.78 - 1.3) | 1.05  (.74 - 1.36) | 1.02***  (.51 - 1.54) | 1.01***  (.51 - 1.50) |
| 6 months | -2.18***  (-2.64 - -1.71) | -2.12***  (-2.56 - -1.67) | -2.13***  (-2.56 -1.69) | -2.15  (-2.64 - 1.66) | -2.25***  (-2.78 - -1.73) | -2.34***  (-2.86 - -1.83) | 1.01***  (0.71 - 1.31) | 1.13***  (.85 - 1.41) | 1.13***  (.85 - 1.41) | 1.04  (.70 - 1.38) | .74**  (.29 - 1.19) | .80***  (.36 - 1.23) |
| 1 month * Accompanied | -1.08**  (-1.71 - -0.45) | -1.14*** ( -1.78 - -.50) | -1.12***  (-1.75 - -.49) | -.95*  (-1.68 - -.21) | -1.24***  (-1.95 - -0.52) | -1.11**  (-1.85 - -0.37) | 0.44*  (0.02 - 0.86) | .35  (-.06 - .76) | .34  (-.06 - .75) | .32  (-.16 - .79) | .33  (-.30 - 0.97) | .46  (-.20 - 1.11) |
| 6 months * Accompanied | -0.96**  (-1.61 - -0.30) | -1.03 **  (-1.70 - -.37) | -1.03**  (-1.68 - -.37) | -.94**  (-1.62 - -.25) | -.89*  (-1.74 - -.04) | -.67  (-1.60 - .25) | 0.68**  (0.25 - 1.12) | .42  (-.01 - .86) | .42  -.01 - .85) | .43  (-.07 - .92) | .97***  (.42 - 1.53) | .80*  (.17 - 1.43) |

* p< 0.05 ** p<0.01 *** p<.001

Table displays regression coefficients and 95% confidence intervals.

IPW= Inverse Probability Weights

n_obs_ represents all observations across all time points included in the model
